# Supplementary material for: Dietary Influence on Systolic and Diastolic Blood Pressure in the TwinsUK Cohort
Source: Nutrients. 2020 Jul 17;12(7):2130. doi: 10.3390/nu12072130 (PMC7400881; doi:10.3390/nu12072130)
Supplement: Supplementary file 1 [file nutrients-12-02130-s001.pdf]

Table S1. Description of dietary indices

Index	Description
**NOVA classification system**	Classifying foods as unprocessed or minimally processed foods, processed culinary ingredients, processed foods and ultra-processed foods, stratifying the sample.
**HEI score**	A measure of diet quality in relation to dietary guidelines for Americans, generates a numerical score of diet quality.
**aHEI score**	As above but further includes foods and nutrients whereby there is evidence to predict risk of chronic disease risk.
**DASH score**	A score developed to capture a diets correspondence to DASH recommendations.
**aMED score**	A score to capture similarities to a Mediterranean diet.
**DQII**	An index developed from the HEI and dietary quality index for cross-cultural comparisons.
**PDI**	A plant-based scoring system. Favours intakes of plant foods and opposes any animal-derived foods.
**uPDI**	As above but unhealthy plant foods such as refined grains, sugar and processed foods contribute the score, but reverse scores are given to animal and healthy plant foods
**hPDI**	Healthy/plant foods are given positive scores and less-healthy plant food and animal foods receive reverse scores

HEI, Healthy Eating Index; aHEI, alternate HEI; DASH, Dietary Approaches to Stop Hypertension; aMED, alternate Mediterranean diet; DQII, Dietary quality index international; PDI, Plant Diversity Index; uPDI, unhealthy PDI; Healthy PDI, hPDI.

Table S2. Descriptive nutrient intakes of TwinsUK sample (*n=3889*)

Nutrient intake	Mean	SD
Alcohol (g)	9.54	13.56
Water (g)	2719.24	780.97
Total nitrogen (g)	13.07	3.64
Protein (g)	80.5	22.46
Fat (g)	69.1	24.78
Carbohydrate (g)	247.97	77.8
Starch (g)	121.49	44.09
Total sugars (g)	123.82	45.92
Glucose (g)	21.61	10.56
Fructose (g)	25.78	13.38
Sucrose (g)	48.56	22.1
Maltose (g)	3.8	2.27
Lactose (g)	20.27	10.6
NSP (g)	20.05	7.67
Saturated fat (g)	25.47	10.43
MUFA (g)	22.75	8.63
PUFA (g)	15.36	6.06
Trans fat (g)	1.66	0.87
Cholesterol (mg)	224.86	93.63
Sodium (mg)	2249.27	785.26
Potassium (mg)	3920.82	1058.97
Calcium (mg)	1096.2	386.02
Magnesium (mg)	340.48	96.4
Phosphorus (mg)	1494.38	421.83
Iron (mg)	12.62	4.23
Copper (mg)	1.52	0.64
Zinc (mg)	9.99	2.92
Chloride (mg)	2607.43	1224.02

**Manganese (mg)**	4.05	1.45
**Iodine (µg)**	214.46	82.34
**Retinol (µg)**	529.55	725.51
**Carotene (µg)**	5037.27	3338.25
**Vitamin D (µg)**	2.52	1.36
**Vitamin E (mg)**	11.08	4.56
**Thiamin (mg)**	1.74	0.57
**Riboflavin (mg)**	2.38	0.88
**Niacin (mg)**	21.51	6.87
**Tryptophan (mg)**	17.06	4.76
**Vitamin B6 (mg)**	2.49	0.78
**Vitamin B12 (µg)**	6.15	3.16
**Folate (µg)**	387.83	148.41
**Pantothenate (mg)**	6.94	11.83
**Biotin (µg)**	47.39	14.7
**Vitamin C (mg)**	157.29	81.48
**Selenium (µg)**	48.34	17.17

Table S3. Nutrient effects on SBP and DBP

Nutrient	SBP				DBP			
	Beta (z-score)	SE (z-score)	P	FDR	Beta (z-score)	SE (z-score)	P	FDR
**Alcohol**	0.564	-0.448	$2.27x10^{-2}$	$2.27x10^{-2}$	0.584	-0.495	$1.62x10^{-4}$	$3.24x10^{-4}$
**Water**	0.359	-0.585	$2.79x10^{-6}$	$5.59x10^{-6}$	0.364	-0.586	$1.94x10^{-2}$	$1.94 x10^{-2}$
**Nitrogen**	-0.842	0.417	$5.16x10^{-2}$	$1.03x10^{-1}$	0.016	0.085	$3.99x10^{-1}$	$3.99x10^{-1}$
**Protein**	0.170	-0.427	$4.71x10^{-2}$	$9.41x10^{-2}$	0.295	-0.480	$2.72x10^{-1}$	$2.72x10^{-1}$
**Total fat**	0.187	-0.426	$7.16x10^{-2}$	$1.43x10^{-1}$	0.318	-0.479	$4.62x10^{-1}$	$4.62x10^{-1}$
**Carbohydrates**	0.193	-0.533	$2.28x10^{-7}$	$4.55x10^{-7}$	0.318	-0.551	$2.98x10^{-2}$	$2.98x10^{-2}$
**Starch**	0.219	-0.517	$6.25x10^{-4}$	$1.25x10^{-3}$	0.348	-0.541	$5.02x10^{-1}$	$5.02x10^{-1}$
**Total sugars**	0.254	-0.524	$4.11x10^{-3}$	$8.22x10^{-3}$	0.312	-0.545	$3.53x10^{-2}$	$3.53x10^{-2}$
**Glucose**	0.375	-0.365	$9.55x10^{-1}$	$9.55x10^{-1}$	0.325	-0.439	$6.48x10^{-1}$	$9.55x10^{-1}$
**Fructose**	0.368	-0.420	$9.91x10^{-1}$	$9.91x10^{-1}$	0.332	-0.476	$6.11x10^{-1}$	$9.91x10^{-1}$
**Sucrose**	0.192	-0.467	$1.88x10^{-2}$	$3.76x10^{-2}$	0.306	-0.507	$2.19x10^{-1}$	$2.19x10^{-1}$
**Maltose**	-1.505	0.462	$3.94x10^{-3}$	$7.88x10^{-3}$	-0.071	0.112	$3.11x10^{-1}$	$3.11x10^{-1}$
**Lactose**	-0.257	-0.379	$1.31x10^{-6}$	$2.61x10^{-6}$	0.085	-0.449	$9.94x10^{-4}$	$9.94x10^{-4}$
**NSP**	0.138	-0.263	$2.53x10^{-1}$	$2.53x10^{-1}$	0.202	-0.370	$2.19x10^{-1}$	$2.53x10^{-1}$
**Saturated fats**	-0.073	-0.279	$2.13x10^{-2}$	$4.26x10^{-2}$	0.322	-0.382	$7.22x10^{-1}$	$7.22x10^{-1}$
**MUFA**	0.035	-0.177	$1.91x10^{-1}$	$3.82x10^{-1}$	0.283	-0.314	$6.19x10^{-1}$	$6.19x10^{-1}$
**PUFA**	0.025	-0.100	$2.57x10^{-1}$	$2.57x10^{-1}$	0.056	-0.265	$1.19x10^{-1}$	$2.38x10^{-1}$
**Trans fats**	-2.530	2.425	$1.21x10^{-1}$	$2.42x10^{-1}$	0.281	1.415	$9.45x10^{-1}$	$9.45x10^{-1}$
**Cholesterol**	0.368	-0.559	$9.63x10^{-1}$	$9.63x10^{-1}$	0.374	-0.569	$5.88x10^{-1}$	$9.63x10^{-1}$
**Sodium**	0.368	-0.584	$7.71x10^{-1}$	$7.71x10^{-1}$	0.366	-0.585	$4.31x10^{-1}$	$7.71x10^{-1}$
**Potassium**	0.361	-0.585	$3.33x10^{-4}$	$6.66x10^{-4}$	0.364	-0.586	$2.28x10^{-2}$	$2.28x10^{-2}$
**Calcium**	0.352	-0.580	$4.69x10^{-4}$	$9.38x10^{-4}$	0.361	-0.583	$4.91x10^{-2}$	$4.91x10^{-2}$
**Magnesium**	0.311	-0.554	$6.73x10^{-3}$	$1.35x10^{-2}$	0.343	-0.565	$8.11x10^{-2}$	$8.11x10^{-2}$
**Phosphorus**	0.346	-0.579	$6.10x10^{-5}$	$1.22x10^{-4}$	0.358	-0.582	$1.31x10^{-2}$	$1.31x10^{-2}$

**Iron**	0.217	0.078	$7.15x10^{-1}$	$7.15x10^{-1}$	0.566	-0.142	$4.71x10^{-1}$	$7.15x10^{-1}$
**Copper**	1.525	3.529	$6.50x10^{-1}$	$6.50x10^{-1}$	1.150	2.180	$6.48x10^{-1}$	$6.50x10^{-1}$
**Zinc**	-0.794	0.625	$1.21x10^{-1}$	$2.43x10^{-1}$	0.061	0.221	$5.41x10^{-1}$	$5.41x10^{-1}$
**Chloride**	0.367	-0.585	$8.73x10^{-1}$	$8.73x10^{-1}$	0.366	-0.586	$2.41x10^{-1}$	$4.82x10^{-1}$
**Manganese**	-1.340	1.050	$9.18x10^{-2}$	$1.84x10^{-1}$	-0.329	0.512	$3.06x10^{-1}$	$3.06x10^{-1}$
**Iodine**	0.286	-0.558	$8.14x10^{-6}$	$1.63x10^{-5}$	0.338	-0.568	$1.55x10^{-2}$	$1.55x10^{-2}$
**Retinol**	0.363	-0.584	$7.29x10^{-2}$	$1.46x10^{-1}$	0.367	-0.585	$8.20x10^{-1}$	$8.20x10^{-1}$
**Carotene**	0.367	-0.587	$6.91x10^{-1}$	$6.91x10^{-1}$	0.367	-0.587	$1.30x10^{-1}$	$2.61x10^{-1}$
**Vitamin D**	-0.263	1.064	$5.37x10^{-1}$	$5.37x10^{-1}$	0.828	0.508	$4.96x10^{-1}$	$5.37x10^{-1}$
**Vitamin E**	-0.179	0.050	$1.66x10^{-1}$	$2.47x10^{-1}$	0.063	-0.163	$2.47x10^{-1}$	$2.47x10^{-1}$
**Thiamin**	-4.936	4.478	$9.05x10^{-2}$	$1.66x10^{-1}$	-2.527	2.794	$1.66x10^{-1}$	$1.66x10^{-1}$
**Riboflavin**	-5.605	2.250	$6.77x10^{-4}$	$1.35x10^{-3}$	-1.808	1.303	$6.28x10^{-2}$	$6.28x10^{-2}$
**Niacin**	0.046	-0.210	$1.69x10^{-1}$	$3.37x10^{-1}$	0.303	-0.335	$6.80x10^{-1}$	$6.80x10^{-1}$
**Tryptophan**	-0.922	0.191	$7.49x10^{-3}$	$1.50x10^{-2}$	0.091	-0.067	$3.92x10^{-1}$	$3.92x10^{-1}$
**Vitamin_b6**	0.273	2.987	$9.66x10^{-1}$	$9.66x10^{-1}$	0.778	1.794	$7.80x10^{-1}$	$9.66x10^{-1}$
**Vitamin_b12**	-0.569	0.185	$5.02x10^{-2}$	$1.00x10^{-1}$	0.202	-0.072	$6.05x10^{-1}$	$6.05x10^{-1}$
**Folate**	0.356	-0.572	$2.45x10^{-1}$	$4.91x10^{-1}$	0.363	-0.577	$5.24x10^{-1}$	$5.24x10^{-1}$
**Pantothenate**	0.481	-0.310	$5.08x10^{-1}$	$5.08x10^{-1}$	0.546	-0.403	$1.17x10^{-1}$	$2.34x10^{-1}$
**Biotin**	-0.289	-0.414	$1.04x10^{-9}$	$2.07x10^{-9}$	0.116	-0.471	$5.10x10^{-4}$	$5.10x10^{-4}$
**Vitamin C**	0.376	-0.561	$6.13x10^{-1}$	$6.13x10^{-1}$	0.376	-0.570	$4.25x10^{-1}$	$6.13x10^{-1}$
**Selenium**	0.415	-0.440	$6.05x10^{-1}$	$6.05x10^{-1}$	0.427	-0.488	$3.31x10^{-1}$	$6.05x10^{-1}$

Table S4. Dietary index's effects on SBP and DBP

Index	SBP			DBP		
	**Beta**	**SE**	**P**	**Beta**	**SE**	**P**
**HEI score**	-0.011	0.033	0.74	-0.008	0.021	0.70
**aHEI score**	-0.037	0.034	0.27	-0.003	0.022	0.88
**DASH score**	-0.109	0.082	0.18	-0.027	0.053	0.61
**aMED score**	0.037	0.173	0.83	0.076	0.113	0.50
**DQII**	0.002	0.047	0.96	-0.002	0.031	0.95
**PDI**	0.060	0.040	0.14	0.020	0.030	0.49
**uPDI**	-0.060	0.030	0.05	0.020	0.020	0.32
**hPDI**	0.040	0.030	0.28	-0.020	0.020	0.42

HEI, Healthy Eating Index; aHEI, alternate HEI; DASH, Dietary Approaches to Stop Hypertension; aMED, alternate Mediterranean diet; DQII, Dietary quality index international; PDI, Plant Diversity Index; uPDI, unhealthy PDI; Healthy PDI, hPDI.

Table S5. Heritability estimates of nutrient intakes

Nutrient Intake	Best Model	A[95%CI]	C[95%CI]	E[95%CI]
**Alcohol (g)**	AE	0.52[0.46;0.58]	0	0.48[0.42;0.54]
**Biotin (µg)**	AE	0.41[0.35;0.46]	0	0.59[0.54;0.65]
**Calcium (mg)**	AE	0.36[0.3;0.41]	0	0.64[0.59;0.7]
**Carbohydrate (g)**	AE	0.53[0.48;0.57]	0	0.47[0.43;0.52]
**Iodine (µg)**	CE	0	0.28[0.23;0.32]	0.72[0.68;0.77]
**Lactose (g)**	AE	0.35[0.29;0.4]	0	0.65[0.6;0.71]
**Magnesium (mg)**	AE	0.45[0.4;0.5]	0	0.55[0.5;0.6]
**Phosphorus (mg)**	AE	0.41[0.36;0.47]	0	0.59[0.53;0.64]
**Potassium (mg)**	AE	0.45[0.4;0.5]	0	0.55[0.5;0.6]
**Riboflavin (mg)**	AE	0.35[0.29;0.41]	0	0.65[0.59;0.71]
**Saturated fat (g)**	AE	0.42[0.36;0.47]	0	0.58[0.53;0.64]
**Starch (g)**	AE	0.42[0.36;0.47]	0	0.58[0.53;0.64]
**Sucrose (g)**	AE	0.35[0.29;0.41]	0	0.65[0.59;0.71]
**Tryptophan (mg)**	AE	0.27[0.21;0.33]	0	0.73[0.67;0.79]
**Water (g)**	AE	0.45[0.39;0.5]	0	0.55[0.5;0.61]

A denotes additive genetic variance, C represents represents common environmental factors and E represents non-shared/unique environmental variance.

Table S6. Heritability estimates of blood pressure

Nutrient Intake	Best Model	A[95%CI]	E[95%CI]
**SBP**	AE	0.54[0.49;0.58]	0.46[0.42;0.51]
**DBP**	AE	0.58[0.53;0.62]	0.42[0.38;0.47]

Abbreviations: SBP, systolic blood pressure; DBP, diastolic blood pressure
